# A study on latency time of fruit and nut allergy in pollinosis patients

**DOI:** 10.1186/2045-7022-1-S1-P112

**Published:** 2011-08-12

**Authors:** Enrique Martín Casañez, Emilio López Cano, Ruiz Marina Dueñas, Rosario González Mendiola, Amanda Rocio González Ramirez, Maria Pilar Lara de la Rosa, Diego Martínez Bóhigas

**Affiliations:** 1 Villarrobledo Hospital, University Hospital Complex of Albacete, Allergology, Albacete, Spain; 2 University Hospital San Cecilio, Degree in statistics, Granada, Spain; 3 University Hospital Complex of Albacete, MSc in Pharmacy, Albacete, Spain; 4 Hospital de la Cruz Roja, Allergology, Madrid, Spain; 5 Fibao-University Hospital San Cecilio, MSc in statistics, Granada, Spain

## Objective

To study the average time elapsed in patients diagnosed of fruit and nut allergy, who previously presented allergy to pollen with rhinoconjunctivitis symptoms and/or asthma. To describe possible influencing factors during the said average time.

## Material and methods

This is a retrospective study conducted with the information collected from 90 patients allergic to fruit or nut for a period of ten months. The following data were collected: sex, age, pollen allergy type and clinical form, type of allergy to fruit and nut and its clinical manifestation and whether latex sensitization was present or not. The analysis was performed using the SPSS 17.0 statistical package. Descriptive and analysis survival techniques have also been used.

## Results

Patients' age ranged from 12 to 65, 58 were women and 32 men. In the patients studied the average time elapsed from pollen allergy diagnosis to the presentation of fruit and nut allergy symptoms were 4.38 years. Average time for women was 4.62 years and 9.36 months less for men. For those who previously presented rhinoconjunctivitis, average time was 2.69 years and those with asthma or both 5 years. For patients allergic to chenopodiaceas average time was 4.72 years, for those allergic to olive pollen it was 4.05 and for gramineae allergic patients 4.52 years. No significant differences were found with respect to the four fruit groups studied using descriptive techniques.

## Conclusions

In patients with fruit and nut allergy, average latency time from pollen allergy diagnosis is 4.38 years. When comparing average time for patients with rhinoconjunctivitis to that for patients with asthma or both conditions, average time is lower in the first group, with 95% confidence level (2.7 years vs 5 years).

